# Impact of dementia on post-hip fracture walking ability: a stratified analysis based on pre-fracture mobility in Swedish cohorts of older adults

**DOI:** 10.1186/s12877-024-05524-x

**Published:** 2024-11-26

**Authors:** Philip D. G. Burenstam Linder, Dorota D. Religa, Fredrik Gustavsson, Maria Eriksdotter, Margareta Hedström, Sara Hägg

**Affiliations:** 1 Department of Clinical Science, Intervention and Technology (CLINTEC), Karolinska Institutet, Stockholm, Sweden; 2 Department of Medical Epidemiology and Biostatistics, Karolinska Institutet, Stockholm, Sweden; 3 Department of Neurobiology, Care Sciences and Society Clinical Geriatrics, Karolinska Institutet, Stockholm, Sweden; 4 Inflammation and Aging Theme, Karolinska University Hospital, Stockholm, Sweden; 5 Trauma and Reparative Medicine Theme (TRM), Karolinska University Hospital, Stockholm, Sweden

**Keywords:** Hip fracture, Dementia, Walking ability, Risk factors

## Abstract

**Background:**

Hip fractures are a major health concern for older adults, often leading to reduced walking ability. Individuals with dementia may experience worse recovery outcomes. This study aims to explore whether dementia is associated with greater declines in walking ability following hip fractures.

**Methods:**

This register study used data from the Swedish Hip Fracture Register, including data on four-months follow-up on walking ability. The register data was linked to information on dementia diagnosis from other national registers prior to the fracture. All patients > 60 years who suffered a hip fracture in Sweden between 2010 and 2018 were included. Binary logistic regression was used to analyze the loss of walking ability after the hip fracture with adjustment for confounding factors. Stratified analyses were done in four groups based on pre-fracture walking ability: *Alone outdoors*, *Assisted outdoors*, *Alone indoors*, and *Assisted indoors*.

**Results:**

The analysis included 59,402 patients with a hip fracture, of which 17% had dementia prior to the fracture. Having dementia was associated with a complete loss of walking ability four months after hip fracture; the multivariable-adjusted odds ratio for complete loss of walking ability in the dementia group, using the non-dementia group as a reference, was 1.60 (95% Confidence Interval [CI] 1.49–1.72. In analyses stratified by pre-fracture walking ability, the odds ratios were 2.34 (95% Confidence Interval [CI] 2.03–2.69) for Alone outdoors, 1.53 (95% CI 1.29–1.81) for Assisted outdoors, 1.41 (95% CI 1.27–1.56) for Alone indoors, and 1.29 (95% CI 1.09–1.51) for Assisted indoors.

**Conclusions:**

This study demonstrates that patients with dementia have a greater risk of complete loss of walking ability. The most notable difference was observed in patients who had high walking ability prior to the fracture. These findings suggest the need for tailored rehabilitation programs and enhanced post-operative care protocols for patients with dementia undergoing hip fracture surgery, particularly for those who had high walking ability before the fracture.

**Supplementary Information:**

The online version contains supplementary material available at 10.1186/s12877-024-05524-x.

## Introduction

Hip fractures are a major public health concern for older adults, associated with a high risk of morbidity and mortality [1, 2]. In Sweden, the lifetime risk of sustaining a hip fracture is estimated to be 23% for women and 11% for men, with a one-year mortality rate following a hip fracture being one in four [3–5]. Among those who suffer hip fractures, an estimated 20% have dementia, while 40% exhibit cognitive impairment [6]. The fracture itself, along with long preoperative fasting and immobilization, has catabolic effects that may negatively impact both postoperative function and complication rates.

Hip fractures frequently result in reduced walking ability and independence. Few patients recover their pre-fracture walking ability and regain the same functionality in activities of daily living [7]. Patients with dementia are even more vulnerable, as cognitive impairment negatively impacts rehabilitation potential, increasing the risk of not regaining pre-fracture walking ability [8, 9]. Furthermore, the motoric-cognitive theory suggests that cognitive and physical function are intertwined and share several mechanisms that may be of importance after a hip fracture [10]. Moreover, dementia is one of the most important risk factors for developing delirium during pre and post fracture ward, which increases the risk of postoperative complications and mortality [11]. Other factors that impede the rehabilitation of walking ability include discharge to long-term care facilities, male sex, and poorer health status as assessed by the American Society of Anesthesiologists (ASA) grade [9].

The impact of dementia on the prognosis of walking ability after a hip fracture remains inadequately understood, primarily due to limitations in previous studies, such as small sample sizes [8, 12] and failure to account for pre-fracture walking ability [9]. These limitations hinder the ability to draw reliable conclusions and emphasize the need for further research to better understand the relationship between dementia and loss of walking ability following a hip fracture.

This study aims to investigate the association between dementia and the loss of walking ability in older adults after a hip fracture. We hypothesize that patients with dementia experience a greater decline in walking ability following a hip fracture compared to those without dementia. To identify whether the impact varies across different levels of pre-fracture walking ability, the analysis is stratified based on pre-fracture walking ability. By obtaining a more comprehensive understanding of the difficulties encountered by patients with dementia, this study will inform future research and assist clinicians in identifying patients at higher risk of reduced walking ability.

## Methods

### Data acquisition

This was a prospective cohort study using data from the Swedish Hip Fracture Register (SHR) between January 1, 2010, and December 31, 2018. The SHR data were linked to the Swedish Registry for Cognitive/Dementia Disorders (SveDem), National Patient Register (NPR), and Swedish Prescribed Drug Register (PDR) to identify as many patients with dementia as possible and the date of their diagnosis. If a case was identified as having dementia in any of the registries between 2007 and 2018 prior to hip fracture, it was classified as having dementia. The Cause of Death register was used to extract the date of death. The linking was conducted using patients’ unique personal identity numbers assigned to every resident in Sweden.

The following data were retrieved from SHR: sex, age, ASA, fracture date, fracture type, residential status, diagnosis of dementia, discharged to, pre-fracture walking ability and walking ability four months after the fracture date. Pre-fracture walking ability and walking ability at the four-month follow-up were categorized into five levels: *Alone outdoors*, *Assisted outdoors*, *Alone indoors*, *Assisted indoors*, or *Unable to walk*. Each level signifies a degree of dependence. Walking ability four months post-fracture was assessed through a questionnaire or via telephone. The question asked was: “How would you assess your walking ability?” with response options corresponding to the aforementioned categories. The rest of the data were provided either by medical records, the patient, next of kin, or nursing staff with patient knowledge from before the fracture.

### Registers and categorization of dementia

The Swedish Hip Fracture Register had an estimated coverage above 80% for the hip fracture population of Sweden these years and contains data on more than 300 000 hip fractures [13] The data were registered in SHR by a hospital administrator using the patients’ medical records at discharge.

The Swedish Registry for Cognitive/Dementia Disorders (SveDem) is a national quality registry which evaluate and follow-up the quality of diagnostics, treatment and care for patients with different dementia disorders [14]. Patients are included in the registry at the date of the dementia diagnosis. The registry includes patients who receive care in specialist memory clinics, primary care, and nursing homes with annual follow-ups.

The National Patient Register (NPR) is a national health data register in Sweden with high levels of validity and completeness that contains information about diagnoses made in inpatient and specialized outpatient care (excluding primary care) [15]. Patients with one of the following International Classification of Diseases (ICD) codes, were considered to have dementia: F0[0–4], F051, G3[0–1], or A810.

The Swedish Prescribed Drug Register (PDR) is a Swedish national registry that contains information on all drugs dispensed on prescription at pharmacies and most drugs dispensed in long-term care facilities [16]. The registry uses the Anatomical Therapeutic Chemical (ATC) classification system, with the N06D code specifically identifying anti-dementia medications.

### Comorbidity and exclusion criteria

The ASA grade system is used to assess a patient’s physical status prior to surgery. It assigns one of six grades, each indicating the severity of underlying medical conditions and overall health status, from ASA 1 (best) to ASA 5 (worse). ASA grade was used as a measure of comorbidity [17]. ASA classification was assessed preoperatively by local anesthesiologists as part of standard preoperative practice.

**Fig. 1:**
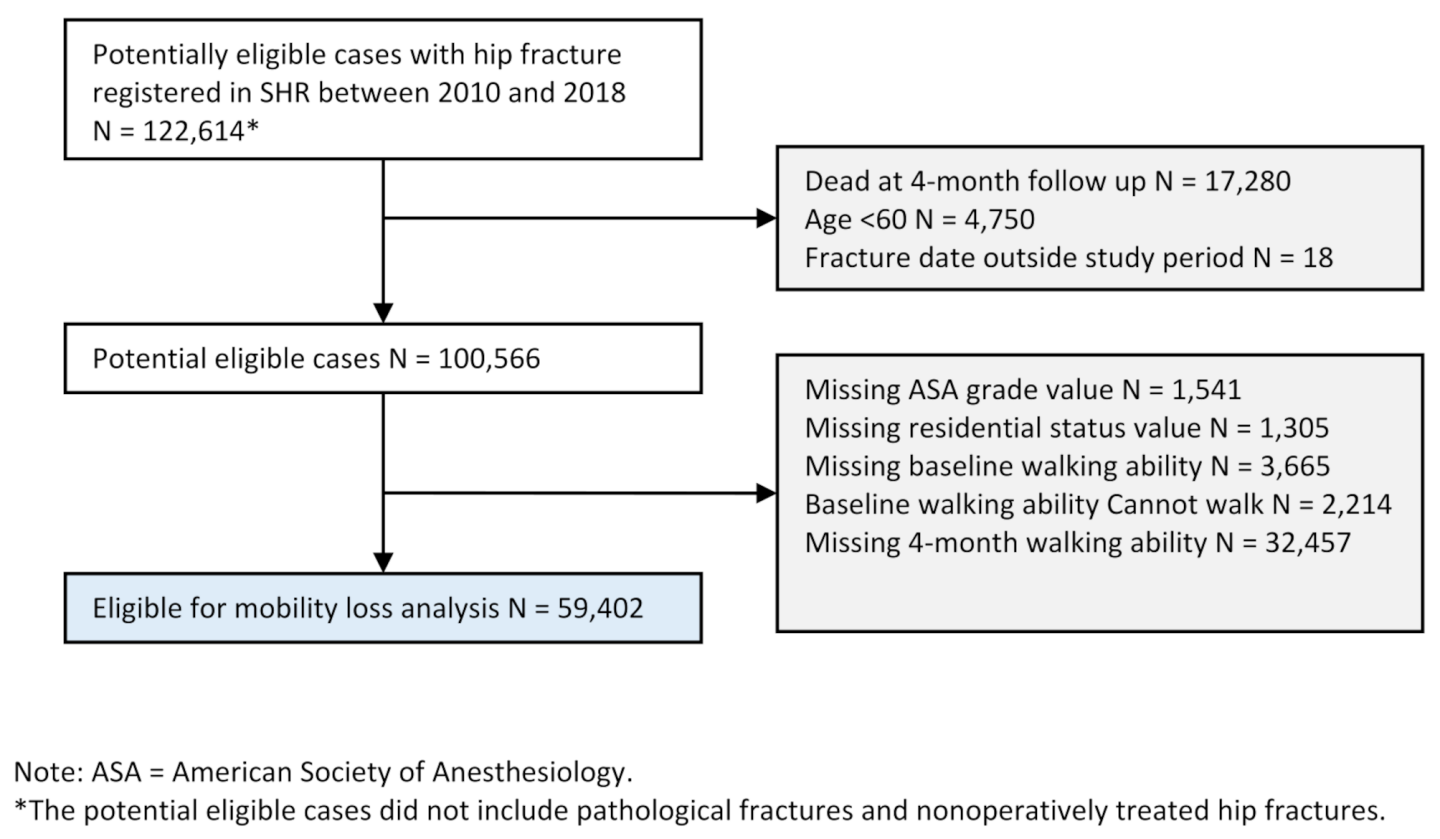
Flow diagram of excluded cases due to missing data or not meeting inclusion criteria

The study included adults who suffered a hip fracture between January 1, 2010, and December 31, 2018. Exclusion criteria were patients under 60 years of age, nonoperatively treated hip fractures, and pathological fractures. Furthermore, patients with missing values for ASA grade, residential status, pre-fracture walking ability, walking ability at follow-up, who had died before follow-up or who could not walk before the hip fracture were excluded. Patients with a pre-fracture walking ability of *Unable to walk* were excluded since there could be no measurable loss of function. Due to missing data on walking ability four months after the hip fracture, 32,457 patients were excluded (Fig. 1). Of the patients lost to follow-up, a higher proportion had an ASA grade of 3 or higher (58% vs. 52%) and dementia (21% vs. 17%) than the patients included in the analysis (Supplementary Table 1, Additional File 1).

### Statistical analysis

The patients were divided into two cohorts: one cohort with a preexisting dementia diagnosis and one without a preexisting dementia diagnosis. Patient characteristics and demographics were presented for the cohorts. Categorical variables are presented as percentages, and continuous variables are presented as the median and interquartile range (IQR). The statistical difference between continuous variables was analyzed using the Wilcoxon rank sum test, and categorical values were analyzed using Pearson’s chi-squared test or Fisher’s exact test for count data. Missing data were handled using complete case analysis. Statistical significance was defined as a two-sided p value less than 0.05, and a 95% confidence interval (CI) was used.

The odds ratio (OR) for complete loss of walking ability, i.e. patients unable to walk at the four-months follow-up, was calculated using binary logistic regression. The patients were stratified into groups based on pre-fracture walking ability level. The ASA grade was reduced to three levels: 1–2, 3 and 4–5 because there were too few patients in each ASA grade after stratification. To account for confounding factors, the multivariable adjustment included the following covariates in the full model: ASA grade, sex, age, residential status, and fracture type. Absolute risk was calculated from relative risks, which were estimated from odds ratios.

All analyses were performed using the statistical programming language R [18].

## Results

### Participant characteristics

There were 59,402 patients who met the inclusion criteria. The distribution in the subgroups based on pre-fracture walking ability was: *Alone outdoors* had 40,582 (68%) patients, *Assisted outdoors* had 4,876 (8%), *Alone indoors* had 10,971 (18%), and *Assisted indoors* had 2,973 (5%) (Table 1). Dementia was identified in a total of 10,174 patients, constituting 17% of the study population (Supplementary Table 2, Additional File 1). Among these, 7,057 patients had documented dementia in the SHR, while an additional 3,528 patients were identified through other registries (NPR, PDR, and SveDem).

Patients with dementia exhibited characteristics indicative of poorer health, such as older age, higher ASA grades, and a greater proportion residing in long-term care facilities. Patients with dementia also had worse pre-fracture walking ability with only 25% able to walk alone outdoors compared to 77% of patients without dementia. Furthermore, patients with dementia had a higher crude risk of complete loss of walking ability after the hip fracture surgery in all pre-fracture walking ability groups: *Alone outdoors (12%* vs. *4%)*,* Assisted outdoors (21%* vs. *13%)*, *Alone indoors (23%* vs. *16%)* and *Assisted indoors (37%* vs. *29%)*.

**Table 1:** The demographic and clinical characteristics of hip fracture patients stratified on pre-fracture walking ability and dementia

Pre-fracture walking ability group:	Alone outdoors			Assisted outdoors			Alone indoors			Assisted indoors		
	**No dementia**, *N* = 38,018 (94%)	**Dementia**, *N* = 2564 (6%)	p value*	**No dementia**, *N* = 3166 (65%)	**Dementia**, *N* = 1710 (35%)	p value*	**No dementia**, *N* = 6509 (59%)	**Dementia**, *N* = 4462 (41%)	p value*	**No dementia**, *N* = 1535 (52%)	**Dementia**, *N* = 1438 (48%)	p value*
Age**	81 (74, 87)	83 (79, 87)	< 0.001	86 (80, 90)	85 (81, 89)	< 0.001	86 (80, 91)	86 (81, 89)	0.001	86 (80, 91)	86 (81, 90)	0.2
Sex			0.3			0.4			< 0.001			0.093
Men	11,769 (31%)	768 (30%)		864 (27%)	487 (28%)		1,750 (27%)	1,054 (24%)		482 (31%)	410 (29%)	
Women	26,249 (69%)	1,796 (70%)		2,302 (73%)	1,223 (72%)		4,759 (73%)	3,408 (76%)		1,053 (69%)	1,028 (71%)	
ASA grade			< 0.001			0.4			< 0.001			0.4
1–2	21,355 (56%)	1,038 (40%)		1,153 (36%)	591 (35%)		2,239 (34%)	1,346 (30%)		394 (26%)	392 (27%)	
3	15,345 (40%)	1,407 (55%)		1,788 (56%)	1,003 (59%)		3,750 (58%)	2,767 (62%)		955 (62%)	889 (62%)	
4–5	1,318 (3.5%)	119 (4.6%)		225 (7.1%)	116 (6.8%)		520 (8.0%)	349 (7.8%)		186 (12%)	157 (11%)	
Walking ability after 4 months			< 0.001			< 0.001			< 0.001			< 0.001
Alone outdoors	21,526 (57%)	538 (21%)		409 (13%)	87 (5.1%)		926 (14%)	161 (3.6%)		163 (11%)	27 (1.9%)	
Assisted outdoors	4,472 (12%)	418 (16%)		726 (23%)	289 (17%)		653 (10%)	326 (7.3%)		122 (7.9%)	71 (4.9%)	
Alone indoors	8,538 (22%)	861 (34%)		984 (31%)	502 (29%)		2,426 (37%)	1,530 (34%)		371 (24%)	237 (16%)	
Assisted indoors	2,011 (5.3%)	431 (17%)		627 (20%)	481 (28%)		1,452 (22%)	1,397 (31%)		431 (28%)	575 (40%)	
Cannot walk	1,471 (3.9%)	316 (12%)		420 (13%)	351 (21%)		1,052 (16%)	1,048 (23%)		448 (29%)	528 (37%)	
Reduced walking ability after 4 months	16,492 (43%)	2,026 (79%)	< 0.001	2,031 (64%)	1,334 (78%)	< 0.001	2,504 (38%)	2,445 (55%)	< 0.001	448 (29%)	528 (37%)	< 0.001
Residential status			< 0.001			< 0.001			< 0.001			< 0.001
Single person household	21,246 (56%)	1,054 (41%)		1,331 (42%)	249 (15%)		2,934 (45%)	694 (16%)		457 (30%)	139 (9.7%)	
Multiple person household	15,499 (41%)	868 (34%)		981 (31%)	383 (22%)		1,590 (24%)	591 (13%)		323 (21%)	164 (11%)	
Long-term care facility	1,273 (3.3%)	642 (25%)		854 (27%)	1,078 (63%)		1,985 (30%)	3,177 (71%)		755 (49%)	1,135 (79%)	
Fracture type			< 0.001			0.4			< 0.001			0.5
Nondisplaced cervical (Garden 1–2)	5,127 (13%)	362 (14%)		329 (10%)	207 (12%)		716 (11%)	591 (13%)		184 (12%)	198 (14%)	
Displaced cervical (Garden 3–4)	15,048 (40%)	1,071 (42%)		1,181 (37%)	649 (38%)		2,351 (36%)	1,697 (38%)		529 (34%)	505 (35%)	
Basicervical	1,199 (3.2%)	95 (3.7%)		116 (3.7%)	57 (3.3%)		245 (3.8%)	162 (3.6%)		52 (3.4%)	57 (4.0%)	
Intertrochanteric (two-part)	7,061 (19%)	449 (18%)		689 (22%)	339 (20%)		1,461 (22%)	929 (21%)		350 (23%)	303 (21%)	
Intertrochanteric (multiple parts)	6,602 (17%)	440 (17%)		599 (19%)	321 (19%)		1,216 (19%)	782 (18%)		303 (20%)	262 (18%)	
Subtrochanteric	2,981 (7.8%)	147 (5.7%)		252 (8.0%)	137 (8.0%)		520 (8.0%)	301 (6.7%)		117 (7.6%)	113 (7.9%)	
Discharged to			< 0.001			< 0.001			< 0.001			< 0.001
Home	20,736 (55%)	637 (25%)		855 (27%)	124 (7.3%)		1,580 (24%)	242 (5.4%)		239 (16%)	49 (3.4%)	
Long-term care facility	14,179 (37%)	1,784 (70%)		2,104 (67%)	1,520 (89%)		4,397 (68%)	3,975 (89%)		1,108 (72%)	1,328 (92%)	
Another hospital or clinic	2,994 (7.9%)	138 (5.4%)		203 (6.4%)	64 (3.7%)		512 (7.9%)	226 (5.1%)		183 (12%)	59 (4.1%)	
Unknown	109	5		4	2		20	19		5	2	

Note: ASA = American Society of Anesthesiology

*Wilcoxon rank sum test; Fisher’s Exact Test for Count Data; Fisher’s Exact Test for Count Data with simulated p value (based on 2000 replicates); **Median (IQR)

### Associations of dementia with post-fracture walking ability across pre-fracture walking levels

Dementia was associated with complete loss of walking ability four months after hip fracture across all pre-fracture walking ability groups (Table 2). The subgroup exhibiting the highest initial pre-fracture walking ability (*Alone outdoors*) was most susceptible to the impact of dementia, with an odds ratio of 2.34 for the loss of walking ability compared to patients without dementia. Conversely, as pre-fracture walking ability worsened, the odds ratio showed a decreasing trend. In the group with the lowest initial walking ability (*Assisted indoors*), the odds ratio was 1.29. However, only comparing these odds ratios could be misleading. The event of complete loss of walking ability occured more frequently with declining pre-fracture walking ability which affected the odds ratio. Consequently, the absolute risk difference variations between the groups was relatively small: 4.6% for *Alone outdoors*, 5.7% for *Assisted outdoors*, 5.3% *Alone indoors*, and 5.4% for *Assisted indoors*.

**Table 2:** Associations with complete loss of walking ability four months after hip fracture stratified on pre-fracture walking ability

Pre-fracture walking ability group:	Alone outdoors			Assisted outdoors			Alone indoors			Assisted indoors		
	**OR**	**95% CI**	*p value*	**OR**	**95% CI**	*p value*	**OR**	**95% CI**	*p value*	**OR**	**95% CI**	*p value*
Dementia	2.34	2.03, 2.69	< 0.001	1.53	1.29, 1.81	< 0.001	1.41	1.27, 1.56	< 0.001	1.29	1.09, 1.51	0.002
Age	1.04	1.03, 1.04	< 0.001	1.01	1.0, 1.02	0.3	1.01	1.00, 1.01	0.15	1.00	0.99, 1.01	0.7
Male	0.59	0.53, 0.66	< 0.001	0.59	0.50, 0.70	< 0.001	0.69	0.62, 0.77	< 0.001	0.66	0.55, 0.78	< 0.001
ASA grade			< 0.001			< 0.001			< 0.001			0.9
1–2	—	—		—	—		—	—		—	—	
3	1.95	1.75, 2.16		1.40	1.17, 1.67		1.16	1.04, 1.29		1.03	0.86, 1.24	
4–5	3.27	2.69, 3.95		1.91	1.41, 2.57		1.50	1.25, 1.79		1.07	0.82, 1.41	
Residential status			< 0.001			0.004			< 0.001			< 0.001
Single person household	—	—		—	—		—	—		—	—	
Multiple person household	1.01	0.90, 1.13		0.97	0.77, 1.21		1.10	0.95, 1.28		0.87	0.66, 1.15	
Long-term care facility	2.79	2.39, 3.25		1.31	1.07, 1.61		1.49	1.32, 1.68		1.42	1.15, 1.76	
Fracture type			< 0.001			0.002			< 0.001			0.013
Nondisplaced cervical (Garden 1–2)	—	—		—	—		—	—		—	—	
Displaced cervical (Garden 3–4)	0.96	0.81, 1.14		0.79	0.61, 1.04		1.00	0.85, 1.18		1.24	0.95, 1.61	
Basicervical	1.57	1.18, 2.06		1.26	0.80, 1.95		1.39	1.05, 1.83		1.86	1.18, 2.90	
Intertrochanteric (two-part)	1.29	1.07, 1.55		0.89	0.67, 1.20		1.21	1.01, 1.44		1.40	1.06, 1.86	
Intertrochanteric (multiple parts)	1.54	1.29, 1.84		1.08	0.81, 1.44		1.39	1.16, 1.67		1.52	1.14, 2.02	
Subtrochanteric	1.82	1.47, 2.25		1.37	0.97, 1.93		1.45	1.16, 1.81		1.60	1.13, 2.29	

Note: Risk factors for patients with complete loss of walking ability at 4 months follow-up stratified based on pre-fracture walking ability

Abbreviations: ASA = American Society of Anesthesiology, OR = Odds Ratio, CI = Confidence Interval

For dementia, no dementia as reference value

For age, age as a continuous value

For male, female as reference value

For ASA grade, ASA 1–2 as reference value

For residential status, single person household as reference value

For fracture type, nondisplaced cervical (Garden 1–2) as reference value

In unstratified analysis with walking ability, while adjusting for pre-fracture walking ability, dementia was associated with complete loss of walking ability four months after hip fracture surgery, OR 1.60 (95% CI 1.49, 1.72) (Supplementary Table 3, Additional File 1). When stratified by sex, patients with dementia are more likely to completely lose walking ability after hip fracture, where this effect by dementia being especially higher in men compared to women in all the pre-fracture walking ability groups (Supplementary Table 4, Additional File 1).

## Discussion

### Summary of findings

The aim of this study was to assess the impact of dementia on the prognosis of walking ability following a hip fracture, with a specific focus on how this impact may vary across different levels of pre-fracture walking ability. Patients with dementia experienced a significantly elevated crude risk of complete loss of walking ability after four months, with 22% experiencing this outcome compared to just 7% of patients without dementia. Additionally, patients with dementia had a higher odds of complete loss of walking ability, irrespective of their pre-fracture mobility status even after adjustments. Notably, those with dementia and the highest pre-fracture walking ability faced a higher odds of complete loss of walking ability compared to their counterparts without dementia. However, the absolute risk differences were similar across all groups.

### Comparing our results to other studies

Previous studies have yielded results consistent with our findings. For example, Tarazona-Santabalbina et al. [19] categorized patients based on the severity of dementia (mild, moderate, and severe) and assessed their ability to walk five meters six months after a hip fracture, with or without the use of assistive devices. They reported odds ratios of 1.33 for mild dementia, 2.05 for moderate dementia, and 2.28 for severe dementia, indicating an increased risk of being unable to walk 5 m. Similarly, Martinez-Carranza et al. [9] found a higher risk of complete loss of walking ability among dementia patients, with an odds ratio of 1.80, using a dataset similar to our study (SHR). These results align with our own findings. Depending on the pre-fracture walking ability, our study also showed odds ratios for complete mobility loss after four months, ranging from 1.29 to 2.34. However, it is important to note that these previous studies did not account for the pre-fracture walking ability, which significantly impacts mobility recovery [20]. Given the notable differences in pre-fracture walking ability between patients with and without dementia (as shown in Table 1), our results may be considered more reliable in this context.

### Differences in the prognosis of hip fracture based on pre-fracture dementia status

We found several differences between individuals with and without dementia following a hip fracture. For instance, at discharge, 40% of patients without dementia returned home, and only 7% of patients with dementia returned to private residences (Table 1). Having a dementia diagnosis often require additional care, and people with dementia more often live in elderly care homes and therefore do not return to their own home. Patients with dementia may also suffer from more motoric dysfunction as suggested by the motoric-cognitive theory. Hence, many discrepancies between hip fracture patients with and without dementia may therefore affect rehabilitation.

Geriatric care significantly reduces mortality, loss of walking ability, and postoperative delirium in older hip fracture patients, especially those with dementia. Multiple studies from Norway and Sweden have highlighted the benefits of geriatric care for patients with hip fractures. Patients treated in a geriatric unit had a significantly lower risk of complete loss of walking ability and lower mortality than those treated in an orthopedic unit [21–23]. In a Swedish randomized control trial, patients with dementia appeared to benefit the most from geriatric care, with an average of 3.2 days of postoperative delirium in the geriatric care group compared to 12.8 days in the orthopedic care group [24]. These findings suggest that healthcare providers should consider the benefits of geriatric care, particularly for patients with dementia, when treating older adults with hip fractures.

### Strengths and limitations

One of the strengths of this study is that it reflects the Swedish hip fracture population, with 80% coverage, providing high generalizability [13, 25]. Hence, our study among other, can provide good background for improving central guidelines for hip fracture management that health care providers should adhere to, which includes rehabilitation protocols in emergency care. Outside of Sweden, the result can best be generalized to other Western countries with similar hip fracture treatment and access.

There are several limitations to this study. The first is the potential misclassification of some patients with dementia as not having the condition. This is because patients in the SHR were recorded as having dementia based on medical discharge records, rather than through a specific dementia assessment. To mitigate this issue, the study enhanced the sensitivity of dementia diagnoses for the hip fracture sample by using multiple registers. A meta-analysis by Seitz et al. [6] found that 20% of hip fracture patients had dementia, which aligns with our finding that 22% of the sample had dementia. However, it is likely that the true proportion of patients with dementia in our study was even higher, as our participants were older than those in the Seitz et al. study.

The second limitation is the 37% loss to follow-up, which may affect the study’s accuracy. Patients lost to follow-up tended to have poorer health (ASA grade III or higher) and a higher prevalence of dementia (Supplementary Table 1, Additional File 1). Due to their worse health status, the study may overestimate the proportion of patients who regain their walking ability underestimate the impact of dementia.

A third limitation is that certain factors were not adjusted for in the study. The study does not adjust for malnutrition, which is known to negatively affect recovery and increase mortality after a hip fracture [23]. This omission could lead to an overestimation of the impact of dementia, as patients with dementia are often more vulnerable to malnutrition. Additionally, cognitive decline can hinder the effective use of walking aids, elevating the risk of complete loss of walking ability in these patients. This study does not account for these differences and other potential differences between the groups, such as the length of hospital stay, occurrence of delirium, incidents of falls, and experiences of depression. However, it is not clear that accounting for these differences would improve the accuracy of the analysis as these factors could be viewed as mediators, rather than being considered confounders.

The fourth and final limitation of the study is the potential misclassification of patient data, which may impact the accuracy of the results. Notably, for patients without dementia in the Assisted indoors group, as many as 11% of patients were later categorized as Alone Outdoors four months after a hip fracture, indicating a substantial improvement in walking ability. Such a significant shift is improbable and likely results from misclassification either at baseline or at follow-up.

## Conclusions and Implications

Patients with dementia are significantly more likely to experience a decline in walking ability after a hip fracture, especially those with high pre-fracture mobility. The study emphasizes the need for targeted rehabilitation strategies and tailored care to mitigate severe mobility loss. Integrating specialized geriatric care and improving rehabilitation protocols in long-term care facilities can enhance recovery outcomes. Clinicians should consider these factors when developing treatment plans to support the rehabilitation and overall well-being of older adults with dementia after hip fracture surgery.

## Electronic supplementary material

Below is the link to the electronic supplementary material.

Supplementary Material 1: Additional tables presenting patient demographics and results

## Data Availability

The datasets generated and analyzed during the current study are not publicly available due to national regulations, please contact the corresponding author with requests.

## Abbreviations

ASA: American Society of Anesthesiologists classification
ATC: Anatomical Therapeutic Chemical
CI: 95% confidence intervals
HR: Hazard ratios
ICD: International Classification of Diseases
IQR: Interquartile range
NPR: National Patient Register
OR: Odds ratio
PDR: National Prescribed Drug Register
SHR: Swedish Hip Fracture Register
SveDem: Swedish Registry for Cognitive/Dementia Disorders
