# Supplementary material for: Impact of dementia on post-hip fracture walking ability: a stratified analysis based on pre-fracture mobility in Swedish cohorts of older adults
Source: BMC Geriatr. 2024 Nov 26;24:970. doi: 10.1186/s12877-024-05524-x (PMC11590525; doi:10.1186/s12877-024-05524-x)
Supplement: Supplementary file 1 — Supplementary Material 1: Additional tables presenting patient demographics and results [file 12877_2024_5524_MOESM1_ESM.docx]

Supplementary Tables

Table S1 **The demographic and clinical characteristics of hip fracture patients lost to follow-up**

|  | ****Lost to follow-up****, N = 32,457 | ****Included****, N = 59,402 | **p-value*** |
| --- | --- | --- | --- |
| Dementia |  |  | <.001 |
| No | 25,779 (79%) | 49,228 (83%) |  |
| Yes | 6,678 (21%) | 10,174 (17%) |  |
| Age** | 83 (76, 88) | 83 (76, 88) | 0.012 |
| Sex |  |  | 0.001 |
| Men | 9,941 (31%) | 17,584 (30%) |  |
| Women | 22,516 (69%) | 41,818 (70%) |  |
| ASA grade |  |  | <.001 |
| 1 | 1,673 (5.2%) | 3,697 (6.2%) |  |
| 2 | 11,846 (36%) | 24,811 (42%) |  |
| 3 | 16,852 (52%) | 27,904 (47%) |  |
| 4 | 2,068 (6.4%) | 2,962 (5.0%) |  |
| 5 | 18 (<0.1%) | 28 (<0.1%) |  |
| Walking ability |  |  | <.001 |
| Alone outdoors | 21,052 (65%) | 40,582 (68%) |  |
| Assisted outdoors | 2,641 (8.1%) | 4,876 (8.2%) |  |
| Alone indoors | 6,575 (20%) | 10,971 (18%) |  |
| Assisted indoors | 2,189 (6.7%) | 2,973 (5.0%) |  |
| Cannot walk | 0 (0%) | 0 (0%) |  |
| Walking ability after 4 months |  |  |  |
| Alone outdoors | 0 (NA%) | 23,837 (40%) |  |
| Assisted outdoors | 0 (NA%) | 7,077 (12%) |  |
| Alone indoors | 0 (NA%) | 15,449 (26%) |  |
| Assisted indoors | 0 (NA%) | 7,405 (12%) |  |
| Cannot walk | 0 (NA%) | 5,634 (9.5%) |  |
| Unknown | 32,457 | 0 |  |
| Residential status |  |  | <.001 |
| Single person household | 16,297 (50%) | 28,104 (47%) |  |
| Multiple person household | 9,438 (29%) | 20,399 (34%) |  |
| Long-term care resident | 6,722 (21%) | 10,899 (18%) |  |
| Fracture type |  |  | <.001 |
| Nondisplaced cervical (Garden 1–2) | 4,374 (13%) | 7,714 (13%) |  |
| Displaced cervical (Garden 3–4) | 12,287 (38%) | 23,031 (39%) |  |
| Basicervical | 930 (2.9%) | 1,983 (3.3%) |  |
| Intertrochanteric (two-part) | 5,714 (18%) | 11,581 (19%) |  |
| Intertrochanteric (multiple parts) | 6,454 (20%) | 10,525 (18%) |  |
| Subtrochanteric | 2,698 (8.3%) | 4,568 (7.7%) |  |
| Note: ASA = American Society of Anesthesiology.  *Fisher's Exact Test for Count Data; Wilcoxon rank sum test; Fisher's Exact Test for Count Data with simulated p-value (based on 2000 replicates); ** Median (IQR). | | | |

Table S2 **The demographic and clinical characteristics of hip fracture patients stratified on dementia**

|  | No dementia, N = 49,228 |  |  |  | Dementia, N = 10,174 | p-value* |
| --- | --- | --- | --- | --- | --- | --- |
| Age** | 82 (75, 88) |  |  |  | 85 (80, 89) | <0.001 |
| Sex |  |  |  |  |  | <0.001 |
| Male | 14,865 (30%) |  |  |  | 2,719 (27%) |  |
| Female | 34,363 (70%) |  |  |  | 7,455 (73%) |  |
| ASA^‡^ grade |  |  |  |  |  | <0.001 |
| 1-2 | 25,141 (51%) |  |  |  | 3,367 (33%) |  |
| 3 | 21,838 (44%) |  |  |  | 6,066 (60%) |  |
| 4-5 | 2,249 (4.6%) |  |  |  | 741 (7.3%) |  |
| Baseline walking ability |  |  |  |  |  | <0.001 |
| Alone outdoors | 38,018 (77%) |  |  |  | 2,564 (25%) |  |
| Assisted outdoors | 3,166 (6.4%) |  |  |  | 1,710 (17%) |  |
| Alone indoors | 6,509 (13%) |  |  |  | 4,462 (44%) |  |
| Assisted indoors | 1,535 (3.1%) |  |  |  | 1,438 (14%) |  |
| Cannot walk | 0 (0%) |  |  |  | 0 (0%) |  |
| Walking ability after 4 months |  |  |  |  |  | <0.001 |
| Alone outdoors | 23,024 (47%) |  |  |  | 813 (8.0%) |  |
| Assisted outdoors | 5,973 (12%) |  |  |  | 1,104 (11%) |  |
| Alone indoors | 12,319 (25%) |  |  |  | 3,130 (31%) |  |
| Assisted indoors | 4,521 (9.2%) |  |  |  | 2,884 (28%) |  |
| Cannot walk | 3,391 (6.9%) |  |  |  | 2,243 (22%) |  |
| Reduced walking ability after 4 months | 21,475 (44%) |  |  |  | 6,333 (62%) | <0.001 |
| Residential status |  |  |  |  |  | <0.001 |
| Single person household | 25,968 (53%) |  |  |  | 2,136 (21%) |  |
| Multiple person household | 18,393 (37%) |  |  |  | 2,006 (20%) |  |
| Long-term care resident | 4,867 (9.9%) |  |  |  | 6,032 (59%) |  |
| Fracture type |  |  |  |  |  | 0.005 |
| Nondisplaced cervical (Garden 1–2) | 6,356 (13%) |  |  |  | 1,358 (13%) |  |
| Displaced cervical (Garden 3–4) | 19,109 (39%) |  |  |  | 3,922 (39%) |  |
| Basicervical | 1,612 (3.3%) |  |  |  | 371 (3.6%) |  |
| Intertrochanteric (two-part) | 9,561 (19%) |  |  |  | 2,020 (20%) |  |
| Intertrochanteric (multiple parts) | 8,720 (18%) |  |  |  | 1,805 (18%) |  |
| Subtrochanteric | 3,870 (7.9%) |  |  |  | 698 (6.9%) |  |
| Discharged to |  |  |  |  |  | <0.001 |
| Home | 23,410 (48%) |  |  |  | 1,052 (10%) |  |
| Long-term care facility | 21,788 (44%) |  |  |  | 8,607 (85%) |  |
| Another hospital or clinic | 3,892 (7.9%) |  |  |  | 487 (4.8%) |  |
| Unknown | 138 |  |  |  | 28 |  |
| Note: ASA = American Society of Anesthesiology.  *Wilcoxon rank sum test; Fisher's Exact Test for Count Data; Fisher's Exact Test for Count Data with simulated p-value (based on 2000 replicates); ** Median (IQR). | | | | | | |

Table S3 **Associations with complete loss of walking ability four months after hip fracture**

|  | OR | 95% CI | p value |
| --- | --- | --- | --- |
| Dementia | 1.60 | 1.49, 1.72 | <.001 |
| Age | 1.02 | 1.02, 1.02 | <.001 |
| Male | 0.62 | 0.58, 0.66 | <.001 |
| ASA grade |  |  | <.001 |
| 1-2 | — | — |  |
| 3 | 1.50 | 1.41, 1.61 |  |
| 4-5 | 2.04 | 1.82, 2.28 |  |
| Residential status |  |  | <.001 |
| Single person household | — | — |  |
| Multiple person household | 1.00 | 0.92, 1.08 |  |
| Long-term care facility | 1.60 | 1.47, 1.73 |  |
| Fracture type |  |  | <.001 |
| Nondisplaced cervical (Garden 1–2) | — | — |  |
| Displaced cervical (Garden 3–4) | 0.99 | 0.90, 1.10 |  |
| Basicervical | 1.51 | 1.27, 1.78 |  |
| Intertrochanteric (two-part) | 1.22 | 1.09, 1.36 |  |
| Intertrochanteric (multiple parts) | 1.42 | 1.27, 1.58 |  |
| Subtrochanteric | 1.59 | 1.40, 1.81 |  |
| Pre-fracture walking ability |  |  | <.001 |
| Alone outdoors | — | — |  |
| Assisted outdoors | 2.55 | 2.31, 2.82 |  |
| Alone indoors | 3.02 | 2.79, 3.27 |  |
| Assisted indoors | 5.46 | 4.93, 6.05 |  |
| Abbreviations: ASA = American Society of Anesthesiology, OR = Odds Ratio, CI = Confidence Interval.  For dementia, no dementia as reference value.  For age, age as a continuous value.  For male, female as reference value.  For ASA grade, ASA 1 - 2 as reference value.  For residential status, single person household as reference value.  For fracture type, nondisplaced cervical (Garden 1-2) as reference value.  For pre-fracture walking ability, alone outdoors as reference value. | | | |

­­­

Table S4 **Risk factors for complete loss of walking ability after 4-months stratified on pre-fracture walking ability and sex**

| 4-month complete loss walking ability; subgroup analysis on males | Alone outdoors | | | Assisted outdoors | | | Alone indoors | | | Assisted indoors | | |
| --- | --- | --- | --- | --- | --- | --- | --- | --- | --- | --- | --- | --- |
| Characteristic | OR | 95% CI | p-value | OR | 95% CI | p-value | OR | 95% CI | p-value | OR | 95% CI | p-value |
| Dementia | 2.68 | 2.15, 3.33 | <.001 | 1.72 | 1.29, 2.29 | <.001 | 1.50 | 1.24, 1.82 | <.001 | 1.33 | 1.00, 1.78 | 0.048 |
| Age | 1.04 | 1.03, 1.05 | <.001 | 1.00 | 0.98, 1.02 | >0.9 | 1.00 | 0.99, 1.02 | 0.6 | 1.00 | 0.99, 1.02 | 0.6 |
| ASA grade |  |  | <.001 |  |  | 0.035 |  |  | 0.048 |  |  | 0.4 |
| 1-2 | — | — |  | — | — |  | — | — |  | — | — |  |
| 3 | 1.74 | 1.48, 2.06 |  | 1.25 | 0.92, 1.71 |  | 1.09 | 0.88, 1.35 |  | 0.78 | 0.55, 1.10 |  |
| 4-5 | 3.03 | 2.28, 3.98 |  | 1.85 | 1.16, 2.93 |  | 1.49 | 1.08, 2.05 |  | 0.79 | 0.48, 1.27 |  |
| Residential status |  |  | <.001 |  |  | 0.6 |  |  | 0.003 |  |  | 0.022 |
| Single person household | — | — |  | — | — |  | — | — |  | — | — |  |
| Multiple person household | 0.91 | 0.77, 1.07 |  | 0.84 | 0.58, 1.22 |  | 0.88 | 0.68, 1.14 |  | 0.87 | 0.55, 1.37 |  |
| Long-term care resident | 2.61 | 2.03, 3.34 |  | 0.88 | 0.60, 1.30 |  | 1.29 | 1.02, 1.63 |  | 1.39 | 0.95, 2.06 |  |
| Fracture type |  |  | 0.002 |  |  | 0.3 |  |  | 0.13 |  |  | 0.5 |
| Nondisplaced cervical (Garden 1–2) | — | — |  | — | — |  | — | — |  | — | — |  |
| Displaced cervical (Garden 3–4) | 0.94 | 0.74, 1.22 |  | 0.85 | 0.55, 1.33 |  | 1.19 | 0.90, 1.60 |  | 1.33 | 0.87, 2.05 |  |
| Basicervical | 1.34 | 0.87, 2.00 |  | 1.22 | 0.59, 2.44 |  | 1.36 | 0.83, 2.19 |  | 1.52 | 0.72, 3.15 |  |
| Intertrochanteric (two-part) | 1.17 | 0.89, 1.54 |  | 0.99 | 0.62, 1.60 |  | 1.40 | 1.02, 1.93 |  | 1.52 | 0.95, 2.44 |  |
| Intertrochanteric (multiple parts) | 1.37 | 1.03, 1.81 |  | 1.13 | 0.69, 1.88 |  | 1.50 | 1.07, 2.10 |  | 1.54 | 0.94, 2.54 |  |
| Subtrochanteric | 1.54 | 1.09, 2.15 |  | 1.57 | 0.82, 2.97 |  | 1.54 | 1.00, 2.35 |  | 1.54 | 0.80, 2.95 |  |
| 4-month complete loss walking ability; subgroup analysis on females |  |  |  |  |  |  |  |  |  |  |  |  |
| Characteristic | OR | 95% CI | p-value | OR | 95% CI | p-value | OR | 95% CI | p-value | OR | 95% CI | p-value |
| Dementia | 2.13 | 1.76, 2.55 | <.001 | 1.45 | 1.17, 1.79 | <.001 | 1.37 | 1.21, 1.56 | <.001 | 1.27 | 1.04, 1.55 | 0.018 |
| Age | 1.04 | 1.03, 1.05 | <.001 | 1.01 | 0.99, 1.02 | 0.3 | 1.01 | 1.00, 1.02 | 0.14 | 0.99 | 0.98, 1.01 | 0.4 |
| ASA grade |  |  | <.001 |  |  | <.001 |  |  | <.001 |  |  | 0.3 |
| 1-2 | — | — |  | — | — |  | — | — |  | — | — |  |
| 3 | 2.09 | 1.83, 2.40 |  | 1.47 | 1.19, 1.83 |  | 1.19 | 1.05, 1.35 |  | 1.15 | 0.93, 1.43 |  |
| 4-5 | 3.42 | 2.59, 4.45 |  | 1.90 | 1.27, 2.81 |  | 1.49 | 1.19, 1.85 |  | 1.24 | 0.89, 1.74 |  |
| Residential status |  |  | <.001 |  |  | <.001 |  |  | <.001 |  |  | <.001 |
| Single person household | — | — |  | — | — |  | — | — |  | — | — |  |
| Multiple person household | 1.11 | 0.95, 1.29 |  | 0.97 | 0.72, 1.30 |  | 1.24 | 1.03, 1.50 |  | 0.85 | 0.59, 1.21 |  |
| Long-term care resident | 2.92 | 2.39, 3.54 |  | 1.52 | 1.20, 1.94 |  | 1.56 | 1.36, 1.80 |  | 1.44 | 1.11, 1.87 |  |
| Fracture type |  |  | <.001 |  |  | 0.024 |  |  | <.001 |  |  | 0.037 |
| Nondisplaced cervical (Garden 1–2) | — | — |  | — | — |  | — | — |  | — | — |  |
| Displaced cervical (Garden 3–4) | 0.98 | 0.78, 1.23 |  | 0.78 | 0.56, 1.09 |  | 0.92 | 0.75, 1.12 |  | 1.15 | 0.83, 1.62 |  |
| Basicervical | 1.79 | 1.22, 2.59 |  | 1.24 | 0.68, 2.18 |  | 1.40 | 1.00, 1.95 |  | 2.13 | 1.21, 3.73 |  |
| Intertrochanteric (two-part) | 1.39 | 1.09, 1.78 |  | 0.86 | 0.60, 1.24 |  | 1.13 | 0.91, 1.40 |  | 1.32 | 0.93, 1.88 |  |
| Intertrochanteric (multiple parts) | 1.66 | 1.31, 2.11 |  | 1.06 | 0.74, 1.51 |  | 1.33 | 1.08, 1.65 |  | 1.47 | 1.03, 2.10 |  |
| Subtrochanteric | 2.03 | 1.54, 2.67 |  | 1.31 | 0.87, 1.97 |  | 1.40 | 1.08, 1.82 |  | 1.58 | 1.03, 2.42 |  |
| Note: Risk factors for patients with complete loss of walking ability at 4 months follow-up stratified based on prefracture walking ability stratified by sex.  Abbreviations: ASA = American Society of Anesthesiology, OR = Odds Ratio, CI = Confidence Interval.  For dementia, no dementia as reference value.  For age, age as a continuous value.  For ASA grade, ASA 1 - 2 as reference value.  For residential status, single person household as reference value.  For fracture type, nondisplaced cervical (Garden 1-2) as reference value. | | | | | | | | | | | | |
